# Acute Popliteal Artery Thrombotic Occlusion Post Total Knee Arthroplasty in a Patient With Antiphospholipid Syndrome: A Report of a Rare Complication

**DOI:** 10.7759/cureus.47054

**Published:** 2023-10-15

**Authors:** Omar A Salem, Mohammed A Alzayer, Mohammed Al Matawah, Mohammed S Alqahtani, Abdullah S Badahdah, Hamad Alshahrani

**Affiliations:** 1 Orthopedic Surgery, King Fahad Specialist Hospital, Dammam, SAU

**Keywords:** endovascular thrombectomy, osteoarthritis, antiphospholipid syndrome, popliteal artery thrombosis, total knee arthroplasty

## Abstract

Acute popliteal arterial thrombotic occlusion following total knee arthroplasty is a rare but serious complication, most of which happens due to blunt trauma during surgery in patients with preexisting peripheral vascular disease. Historically, popliteal artery thrombosis has been approached only by open surgery. In this report, we describe a case of acute thrombotic occlusion of the popliteal artery occurring immediately after total knee arthroplasty in a patient who was presumed healthy and found to have antiphospholipid syndrome and was successfully managed by mechanical endovascular thrombectomy.

## Introduction

Total knee arthroplasty (TKA) is a common surgical procedure for end-stage knee osteoarthritis (OA). It is one of the most clinically successful and cost-effective medical procedures developed during the last ½ century with approximately 1.8 million procedures performed annually worldwide [1,2].

The overall complications following TKA are rare with an incidence rate ranging from 1.65 to 11.3% [3,4]. Some of the common complications after TKA include infection, dislocation, fracture, and deep venous thrombosis [5,6]. Vascular injury is a very rare complication with a reported incidence of 0.03-0.17% [7].

The popliteal artery is the most common injured vessel during TKA. This is due to its close proximity to the knee joint. It is estimated to be around 2.7-9.7 mm from the posterior capsule depending on the knee position and its level making it vulnerable to injury [8].

Acute limb ischemia following TKA is a limb-threatening condition mandating emergent revascularization. Delay for a few hours can significantly increase morbidity. Revascularization procedures can be done either open or endoscopic. The choice of intervention should be studied carefully and depends on the type and extent of arterial injury and the presence of preexisting disease vessel. Recent reports showed endoscopic intervention is effective in the treatment of popliteal artery injury [9]. Simple endoscopic thrombectomy is not always feasible and the vascular surgeon needs to be prepared for open surgical intervention [10,11].

Patients with a prior history of peripheral vascular disease, diabetes mellitus, hypertension, claudication, and previous carotid, coronary, aortic or peripheral vascular surgery are at increased risk of developing arterial injury during TKA [11]. In the absence of risk factors, less-common causes should be considered such as vasculitis, hypercoagulability disorders or autoimmune disease.

Antiphospholipid syndrome (APS) is a systemic autoimmune disorder characterized by recurrent arterial and/or venous thrombosis. On its own, it is known to induce acute limb ischemia. The incidence of lower limb arterial thrombosis is 4.3% in patients with APS [12]. Although it is not common it could lead to serious complications including acute limb ischemia and amputation [12-14].

In this paper, we present a rare case of popliteal artery thrombosis that was diagnosed immediately post TKA in a patient who was presumed healthy but was found to have antiphospholipid syndrome that was treated successfully in a timely fashion with minimally invasive mechanical endovascular thrombectomy.

## Case presentation

Our patient is a 54-year-old female who was admitted to our facility for right TKA. Her overall health condition was very good with an unknown past medical history of chronic illnesses. She denied any history of previous thromboembolism, polyarthritis or abortions. Her only concern was bilateral advanced knee osteoarthritis that was causing her chronic disabling pain for the last 10 years interfering with her daily activity and refractory to non-surgical treatment. Other than the obesity, her general examination, vital signs and lab investigation were all within normal limits. Her weight was 78 with a BMI of 32.5. A local knee examination showed varus deformity of both of her knees with medial midline joint tenderness. Her range of motion was from 10 to 100 degrees both passively and actively. Her distal pulses were palpable with no signs of peripheral vascular disease in both of her lower limbs.

**Figure 1:**
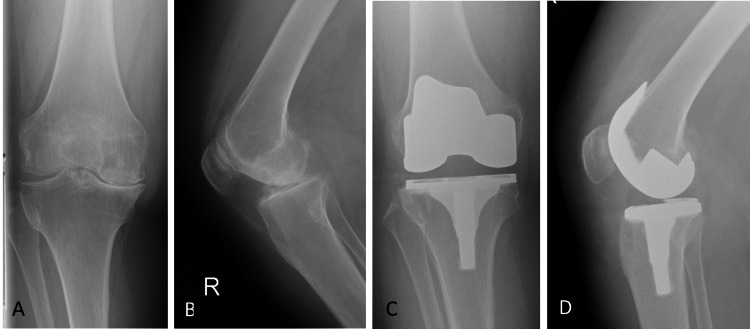
Radiographic images of the right knee. (A and B) pre-operative AP and lateral images showing end-stage knee OA. (C and D) showing post-operative AP and lateral images with well-position implants. OA: Osteoarthritis; AP: Anteroposterior

The patient underwent standard successful right posterior stabilized TKA using the Genesis II TKA system (Smith & Nephew; Memphis, TN, USA) under combined spinal/epidural anesthesia and through a medial parapatellar approach. The total operative time was 65 minutes. A tourniquet was used during the surgery and inflated to 285 mmHg during the whole surgery. Postoperative images showed excellent alignment and fixation of the tibial and femoral implants (Figure 1). Immediate after surgery routine distal neurovascular examination showed diminished distal pulses. Both dorsalis pedis artery and posterior tibial artery were not palpable nor audible on Doppler. Her right foot was pale and cold with no identifiable signal when a pulse oximeter was placed on her right foot.

**Figure 2:**
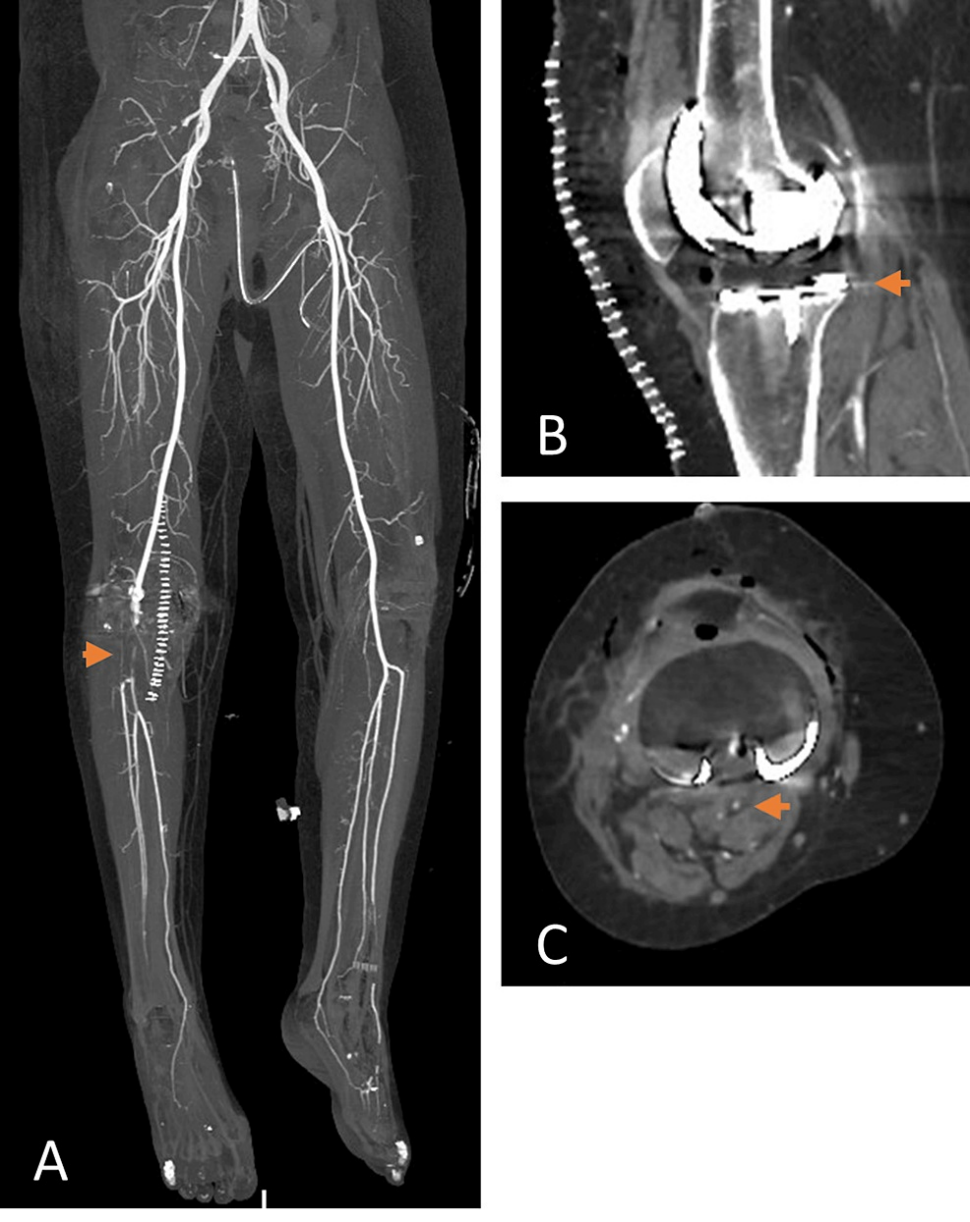
(A-C) Coronal, sagittal and axial images of computed tomography angiogram showing 6 cm thrombotic occlusion of the right popliteal artery extending to TP trunk. TP: Tibioperoneal

The vascular surgery team was urgently involved and they confirmed the clinical findings of acute right lower limb ischemia and a heparin infusion was started immediately. She was then shifted to the radiology department for an emergent CT-angiogram which showed a fairly long segment of complete thrombotic occlusion involving the popliteal artery measuring approximately 6 cm and partially occluding the tibioperoneal trunk (TP trunk). The posterior tibial artery, anterior tibial artery and peroneal artery were opacified with faint attenuation at the level of the ankle (Figure 2).

Options of open surgical thrombectomy and endovascular mechanical thrombectomy were discussed between the orthopedic surgery team, vascular surgery team and intervention radiology team. The patient and her family were involved in discussion and decision-making. An agreement was made to perform the emergent endovascular mechanical thrombectomy.

**Figure 3:**
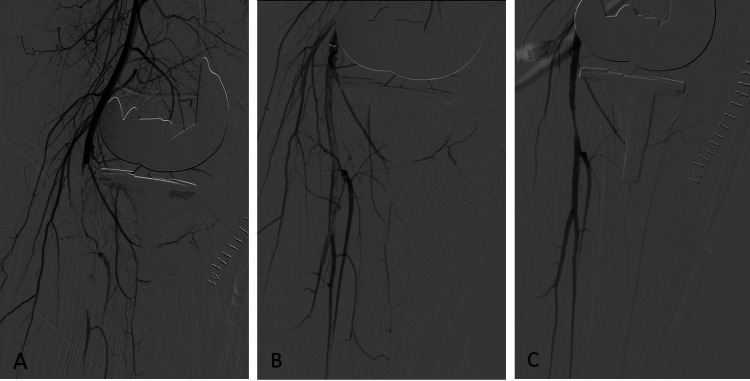
Endovascular treatment with prolonged balloon inflation of occluded popliteal artery. (A) Angiogram showing a total occlusion of the popliteal artery with angiographic presence of thrombus extending to the level of TP trunk. (B) Endovascular thrombectomy via Penumbra Indigo Aspiration System. (C) Restoration of blood flow through the popliteal artery after thrombectomy with good opacification of the three below-knee vessels with presumed area of local spasm. TP: Tibioperoneal

In the intervention radiology department, an 8-Fr sheath was inserted in the right superficial femoral artery. The angiogram showed a long segment of the popliteal artery filling defect extending into the TP trunk. Successful endovascular thrombectomy was achieved via Penumbra Indigo Aspiration System (Penumbra Inc., Alameda, CA, USA). This restored the antegrade blood flow with good opacification of the three below-knee vessels with a presumed area of local spasm (Figure 3). The foot became pink and warm again. Her distal pulses were palpable and the pulse oximeter in the right foot reading was 100%.

The following five days the patient was kept on heparin infusion and was monitored closely. Her foot remained pink and warm. Her distal pulses were both palpable and audible on Doppler. She was then shifted to therapeutic subcutaneous Enoxaparin (1 mg/kg twice daily) which she continued for a total of three months.

The patient resumed her medical and physical therapy treatment the next day of surgery as per post-operative TKA protocol with no delay. She was discharged on the sixth day with a stable and well-perfused right foot, with routine follow-up in the outpatient clinic. Her outcome was good with a good post-operative range of motion and pain control on her one-year post-operative follow-up.

Since the patient had no identifiable risk factors other than obesity, hematology and rheumatology specialists were consulted for further assessment of possible vasculitis, hypercoagulability disorders or autoimmune disease.

Upon further assessment, the patient mentioned that she had bilateral small hand joint pain that started one month back, with morning stiffness lasting around 10 minutes. She gave a history of facial redness increasing with heaters and sun exposure, and infrequent (around every six months) oral ulcers for three years (no scarring), painful, with spontaneous resolution but no genital ulcer. She has diffused hair loss for two years. She had no eye manifestations, no previous thromboembolism nor Raynaud phenomenon. She had no history of bleeding from any orifices (no gum bleeding, epistaxis or GI bleeding) or ecchymosis or petechiae. She denied any family history of hematological or rheumatological diseases. She is a mother of seven kids with no history of abortions.

Laboratory workup revealed a negative lupus anticoagulant but a positive antinuclear antibody (ANA) 1:160, anticardiolipin IgM and β2-glycoprotein IgM on more than two occasions, which confirmed the diagnosis of APS.

It was a challenge to say how much antiphospholipid antibodies and/or surgery have contributed to the event. A decision was made to initiate prophylactic apixaban 2.5 mg po bid plus continuing aspirin is a reasonable option in this case.

## Discussion

Vascular injuries during TKA are extremely rare with very limited reported cases in the literature. The overall incidence is estimated to be between 0.03 and 0.17% [7]. It is thought that it is related to its close proximity to the knee joint and the fact that most of the patients requiring this procedure are elderly with preexisting chronically diseased vessels. Wilson et al. reported that almost 33% of arterial injuries following orthopedic surgery were found to have preexisting atherosclerotic disease in the injured arterial segments [15].

Popliteal artery injury during TKA can be either a direct injury or an indirect injury. A direct arterial injury can be caused by a penetrating sharp object such as during pin or Hohmann placement or by a saw when performing bone cuts. Indirect arterial injuries are believed to be more common [16,17]. Several mechanisms have been hypothesized including: (1) popliteal artery tenting during hyperflexion or hyperextension when performing the bone cuts and implantation, (2) stretching of the artery after surgery in patients with significant preoperative fixed flexion deformity of significant varus/valgus deformity [16], (3) indirect injury from tourniquet inflation.

Several authors believe that the use of a tourniquet during surgery plays a significant role. They argued that in almost all reported cases of popliteal artery injury during TKA a tourniquet was used [17,18]. Authors believe that the tourniquet can induce arterial injury through several mechanisms. It applies excessive external compression force to the superficial femoral artery and subsequently causes membrane damage and/or rupture of atheromatic plaque in a previously diseased artery. Such injury can induce platelet activation and subsequent thrombosis or plaque embolization [19,20]. Authors also believe that the pressure applied to the artery can cause stasis which can induce arterial thrombosis, especially in a high-risk patient with hypercoagulability. Another supposed mechanism is tourniquet inflation anchors in the proximal arterial segment which can cause the artery to stretch during manipulation and make it more vulnerable to injury. Although there is no clear evidence to support such adverse effects of tourniquet use, several authors advised against its use in patients with pre-existing peripheral vascular disease [6,16,21,22].

There are six recognized types of arterial injury: (1) Laceration, (2) transection, (3) arteriovenous fistula formation, (4) pseudoaneurysm or aneurysm formation, (5) spasm and (6) contusion [10,23]. Regardless of the type of arterial injury, the most common presentation is acute limb ischemia from subsequent arterial thrombosis [8]. Signs and symptoms include pain, a cold and pale foot, delayed capillary refill and absent pulses even on Doppler. Frequent use of regional anesthesia has been shown to cause delay in initial recognition and suppress the patients’ symptoms. Keeping a high level of suspension and frequent reassessment is important to prevent delayed recognition [14]. Patients can also present with excessive bleeding or hematoma formation after a direct penetrating injury which causes vessel laceration or transection. Other presentations include localized posterior knee swelling, pulsatile mass and bruits and thrills which are common in cases of arteriovenous fistula, aneurysm and pseudoaneurysm.

Late presentation was not uncommon. Several authors reported delayed presentation from a few days up to several months following surgery. Most of those patients showed no initial manifestations of vascular injury with documented palpable distal pulses [15,24,25]. Most of which were found to have aneurysm/pseudoaneurysm. Late presentations can be misleading and diagnostically challenging. Some of the reports showed delays in reaching for diagnosis and suffering from unfavorable outcomes such as compartment syndrome or gangrene. Hozack et al. reported a 70-year-old male with a popliteal artery pseudoaneurysm that was diagnosed five months following TKA [16].

The risk of injury is higher in patients with a history of peripheral vascular disease [8,11]. Atherosclerotic vessels are narrow, stiff and more prone to injury during manipulation. Other risk factors include diabetes mellitus, hypertension, renal failure, obesity, cancer and previous carotid, coronary aortic disease, renal failure, and coagulopathy [5,11,16]. Previous knee surgeries were also shown to increase the risk of popliteal artery injury [11,25]. This might be due to excessive scar formation and soft tissue contraction which can cause indirect injury during manipulation or extensive soft tissue release [8,10]. Our patient had neither of any of these risk factors. She had no cardiac or vascular diseases. There was no indication for pre-operative vascular surgery consolation or any indication for other pre-operative vascular evaluation other than examining the distal pulses and capillary refill which all were normal in our patient. Further and detailed assessment and workup were mandated. For this reason, rheumatology and hematology specialists were consulted and showed positive antiphospholipid antibodies. To our knowledge, there are no reported cases of acute limb ischemia following TKA in a patient with antiphospholipid syndrome.

Antiphospholipid syndrome (APS) is a systemic autoimmune disorder that increases the risk of developing blood clots. It is often associated with recurrent fetal loss, thrombocytopenia, and elevated antiphospholipid antibodies. Lower limb manifestations include deep vein thrombosis (38.9%), superficial thrombophlebitis (19.8%) and arterial thrombosis (2.7%). Although arterial thrombosis is rare it carries devastating complications including lower limb amputation [12-14].

Virchow’s triad describes three elements that contribute to the development of thrombosis including stasis, endothelial injury and hypercoagulability. We believe that our patient developed all three elements that led to the development of popliteal artery thrombosis - stasis following tourniquet inflation, indirect popliteal artery injury during knee manipulation and hypercoagulability from the presence of anti-phospholipid antibodies [26].

Arterial injury following TKA has traditionally been managed through open surgery, however, recent studies showed promising results with endovascular strategies. Endovascular treatment has the advantage of avoiding general anesthetics with less local morbidity than open surgery [7,24,27,28]. The type of endoscopic intervention depends on the type of arterial injury. Several options are available including pharmacological thrombolysis or mechanical thrombectomy in cases of thrombosis. In cases with pseudoaneurysm angioplasty with balloon dilatation or stent application, and coil embolization can be used [10,24].

Open surgery should still be considered if endoscopic treatment has failed or when the arterial injury is not amenable to endoscopic treatment such as in cases of large vessel laceration or transection requiring open repair [10,24,29]. Vascular surgeons should always be ready for open intervention. Open surgical methods include thrombectomy, open repair of the vessel, excision of the damaged part followed by end-to-end anastomosis, in case of arterial tear or pseudoaneurysm, arterial bypass, or above-the-knee amputation. Some patients required fasciotomy due to delayed vascular repair [19,29]. Our reported case underwent a simple endovascular thrombectomy which successfully regained the blood flow. No further intervention was needed since no arterial injury was found nor vessel disease.

Although vascular injury during TKA carries very devastating and catastrophic consequences with an overall morbidity of 42% and mortality of 7% [8]. They include compartment syndrome, periprosthetic infection, permanent neurological injury, amputation and even death [15,18,19,30]. Reperfusion within the first six hours, so-called “the golden hours” following the injury, is thought to significantly improve the outcome [7,24]. Recent reports showed more favorable outcomes [15,22]. This might be due to improvements in diagnostic modalities and modes of intervention including endoscopic treatment [7,11,17,24].

## Conclusions

So far there are no clear guidelines to help identify or prevent this serious complication. We believe that the identification of patients at risk might guide a more cautious treatment approach or even defer surgery. Further vascular assessment in patients with significant peripheral arterial insufficiency is advisable to understand the extent of the disease before proceeding to surgery. Regardless of the cause of arterial thrombosis early identification and intervention is the key to prevent unfavorable outcomes. Routine monitoring of distal circulation following TKA is crucial. Once suspected, urgent vascular consultation must be done.

The choice of intervention should be studied carefully and discussed between different teams. Endoscopic methods have been proven to be safe and effective in the management of popliteal artery injury during TKA with lower local morbidity than surgical thrombectomy. To our knowledge, this is the only reported case of popliteal artery thrombosis following TKA in a patient with APS. In patients with no obvious risk factors who develop popliteal artery thrombosis, we recommend further assessment by hematology and rheumatology specialists in order to exclude other rare blood clotting disorders.
